# Global research trends in bone tumors: a bibliometric analysis of medical imaging

**DOI:** 10.3389/fonc.2025.1579339

**Published:** 2025-05-21

**Authors:** Yi Zhou, Kaixiang Yang, Wei Cui

**Affiliations:** Department of Radiology, Wuhan Fourth Hospital, Wuhan, China

**Keywords:** bone tumor, medical imaging, bibliometric analysis, osteosarcoma, artificial intelligence

## Abstract

**Background:**

Bone tumors, due to their high rate of misdiagnosis, pose significant clinical challenges in diagnosis and treatment. Medical imaging plays a critical role in the accurate detection, staging, and monitoring of these tumors. Understanding global research trends in this area is crucial to advance diagnostic techniques and therapeutic strategies.

**Methods:**

This study performed a comprehensive bibliometric analysis of publications related to bone tumors and medical imaging from 1995 to 2024. Data were retrieved from the Web of Science Core Collection, and keyword co-occurrence analysis, citation patterns, and publication trends were examined to identify research hotspots and emerging trends.

**Results:**

Our analysis shows a steady increase in the number of publications over the past two decades, with the USA leading with 1,258 publications. The University of Texas System ranks first among institutions with 268 publications, while *Skeletal Radiology* has published the most articles in this field, with 232 publications. Asif Saifuddin is the most prolific author, having published 26 papers. Key research themes include advancements in imaging modalities, bone metastasis, and artificial intelligence (AI) in imaging. Emerging research hotspots include multimodal imaging studies and AI-assisted diagnosis, which are expected to be key areas of future research.

**Conclusion:**

This bibliometric study provides a comprehensive overview of medical imaging research in bone tumors. Multimodal imaging approaches and AI-driven tools for early detection, treatment monitoring, and personalized therapy present promising pathways to enhance patient care in the management of bone tumors.

## 1 Introduction

Bone tumors, including both primary and metastatic types, commonly present with symptoms such as localized pain, swelling, functional impairment, and fractures (1, 2). Primary bone tumors include osteosarcoma, chondrosarcoma, and Ewing sarcoma, with osteosarcoma and Ewing sarcoma showing relatively higher incidence around the age of 20, while chondrosarcoma is more common in the elderly population (3). Metastatic bone tumors are frequently seen in the late stages of malignancies such as breast cancer, lung cancer, and prostate cancer, and constitute a significant part of clinical bone tumor cases. Once cancer spreads to the bones, it becomes difficult to cure and may lead to various other complications (4). Although the incidence of bone tumors is relatively low compared to other cancers, their diverse clinical presentations often lead to misdiagnosis. Early detection and accurate diagnosis are crucial for improving patient prognosis (5). In recent years, research on bone tumors has made significant progress in both basic and clinical medical fields (6, 7). With advancements in molecular biology, immunology, and genomics, researches on the pathological mechanisms of bone tumors (8, 9), molecular biomarkers (10), targeted therapies (11) and immunotherapies (12) have become more in-depth. Notably, advances in medical imaging have driven the early diagnosis and treatment of bone tumors, becoming an essential tool in current clinical practice (13).

Modern imaging technologies, particularly high-resolution magnetic resonance imaging (MRI), computed tomography (CT), and positron emission tomography (PET), have enabled more precise observation of the morphological features, diffusion patterns, and relationships with surrounding tissues of bone tumors, providing reliable diagnostic support for clinical practice (13, 14). For instance, X-ray imaging and CT scans can assess bone destruction, periosteal reactions, and sclerosis of lesions (15). Ultrasound has been reported as a valuable adjunctive tool in the assessment of primary bone tumors, primarily due to its capability to visualize adjacent soft tissue structures and vascular flow without being affected by metal artifacts (16). Nonetheless, its diagnostic utility is limited in evaluating intraosseous components, given the restricted penetration of ultrasound waves through cortical bone (17). MRI can evaluate bone tissue characteristics, tumor extent, and reactive areas, aiding in distinguishing malignant from benign bone lesions (18). The introduction of PET allows for more accurate detection and localization of bone metastases in cancer patients (19). Recently, radiomics and computer-aided diagnostic systems have become cutting-edge fields in bone tumor diagnosis. For example, Sun et al. developed a clinical model combined with radiomics to distinguish between benign and malignant bone tumors, achieving a high degree of accuracy (20). Zhao et al. created a machine-learning model based on CT scans to detect bone tumors metastatic from breast cancer (21).

Bibliometrics, as a quantitative analysis tool, is increasingly being applied by scholars to assess the research status and development trends in various fields (22, 23). By statistically analyzing various information in the literature, bibliometrics provides strong support for identifying research hotspots, development directions, and academic exchange patterns (24). Although some studies have explored related imaging topics, there is currently a lack of comprehensive analysis specifically focusing on bone tumor imaging. To date, no study has systematically applied bibliometric methods to assess the global research trends in the field of medical imaging for bone tumors. This study aims to fill this gap. This research applies bibliometric methods to perform an in-depth analysis of literature related to bone tumor medical imaging from 1995 to 2024, comprehensively revealing the current state of research, emerging trends, and future directions in this field.

## 2 Methods

### 2.1 Data extraction

**Figure 1:**
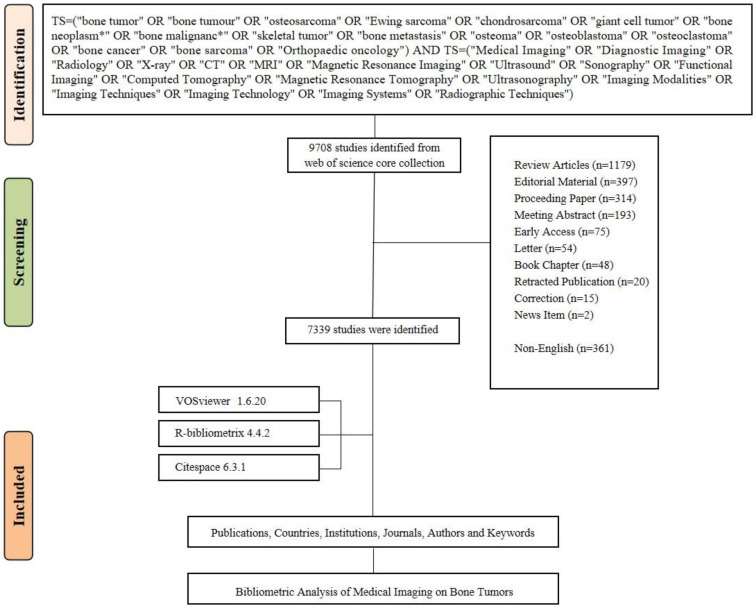
Flowchart of the study.

Given the advantages of the Web of Science Core Collection (WoSCC), which offers rich functionality and high-quality data (23), this study uses this database for literature analysis. The WoSCC database was selected because of its comprehensive coverage of high-quality, peer-reviewed research, including a diverse range of journals across various disciplines. While other databases such as PubMed or Scopus also provide valuable information, WoSCC offers a more extensive set of citation data, which is crucial for conducting robust bibliometric analyses (25). The specific search formula is as follows: TS=(“bone tumor” OR “bone tumor” OR “osteosarcoma” OR “Ewing sarcoma” OR “chondrosarcoma” OR “giant cell tumor” OR “bone neoplasm*” OR “bone malignanc*” OR “skeletal tumor” OR “bone metastasis” OR “osteoma” OR “osteoblastoma” OR “osteoclastoma” OR “bone cancer” OR “bone sarcoma” OR “Orthopaedic oncology”) AND TS=(“Medical Imaging” OR “Diagnostic Imaging” OR “Radiology” OR “X-ray” OR “CT” OR “MRI” OR “Magnetic Resonance Imaging” OR “Ultrasound” OR “Sonography” OR “Functional Imaging” OR “Computed Tomography” OR “Magnetic Resonance Tomography” OR “Ultrasonography” OR “Imaging Modalities” OR “Imaging Techniques” OR “Imaging Technology” OR “Imaging Systems” OR “Radiographic Techniques”). We performed a topic search using these keywords, covering the period from 1995 to 2024. This time frame was selected based on preliminary retrieval results, which indicated that very few relevant articles were found prior to 1995, with publication numbers beginning to increase significantly from 1995 onwards. To minimize errors caused by database updates, all data were downloaded on January 14, 2025. Duplicate records were removed, and only documents of the “article” type published in English were included, excluding reviews, letters, and conference abstracts. The full records and references were exported in “plain text” format for subsequent analysis. **Figure 1** illustrates the detailed analysis workflow.

### 2.2 Data analysis

The bibliometric analysis tools used in this study include the Bibliometrix R package (version 4.4.2), VOSviewer (version 1.6.20), and CiteSpace (version 6.3.1). The Bibliometrix R package was used primarily for quantitative analysis (26) to assess and visualize indicators such as country/region and institution distribution, journal trends, and author influence. VOSviewer is a powerful tool for co-authorship and co-occurrence analysis (27). Specifically, we used VOSviewer to reveal the collaborative relationships among authors, countries, and institutions, as well as to illustrate the interrelationships among various keywords. CiteSpace is a robust citation analysis and visualization tool (28). We used CiteSpace to identify keywords with a significant citation burst over a defined period. The parameters were set to include time slices from 1995 to 2024, with each slice representing one year.

## 3 Results

### 3.1 An overview of publications

**Figure 2:**
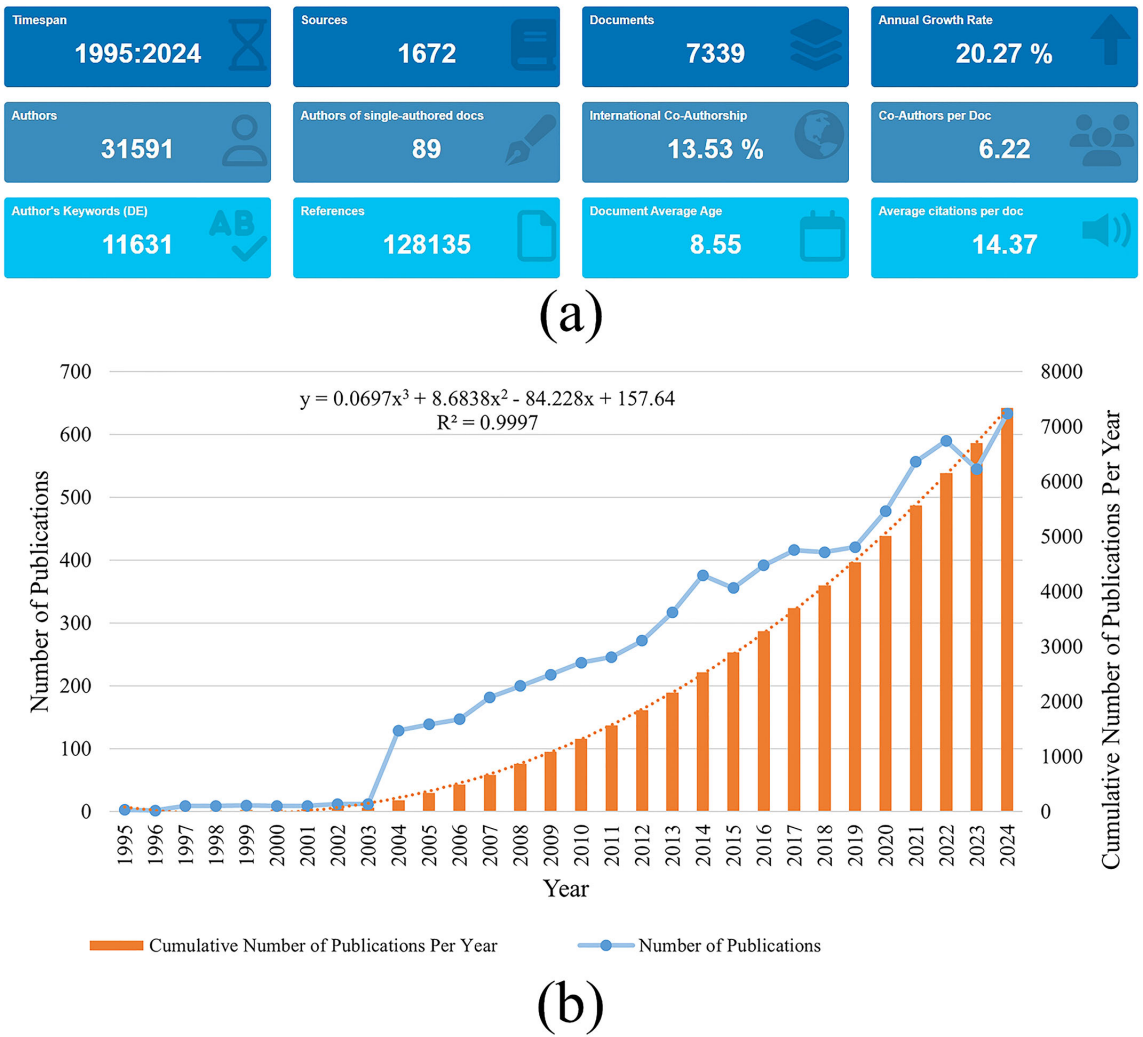
**(a)** Overview of publications in bone tumor imaging research. **(b)** The annual number and the cumulative number of publications.

**Figure 2** provides a comprehensive overview of the research trends in bone tumors and medical imaging. A total of 7,339 documents were published from 1995 to 2024, contributed by 31,591 authors. Notably, a proportion of publications (13.53%) involve international co-authorship, highlighting the extent of global collaboration. On average, each document features 6.22 co-authors, emphasizing the collaborative nature of research in this area. The cumulative number of references stands at 128,135, underlining the extensive citation and cross-referencing within the literature. The average document age is 8.55 years, suggesting that the majority of research in this field is relatively recent. Additionally, an annual growth rate of 20.27% highlights the rapid expansion of the field over the past three decades (1995–2024). In **Figure 2b**, the data reveals a significant increase in the number of publications from the early 2000s, with a particularly steep rise in the last decade. The growth pattern follows a cubic trend, as indicated by the fitted equation and the high R² value of 0.9997. The cumulative number of publications has accelerated significantly since 2015, indicating an expanding body of research in this field. Notably, the number of publications peaked in 2024, marking the highest level of research activity in the field. This surge in 2024 reflects the growing research enthusiasm and highlights the increasing focus on bone tumors and medical imaging as key areas of scientific inquiry.

### 3.2 Analysis of countries

**Table 1** presents the bibliometric analysis of bone tumor research in medical imaging, revealing the contributions of the top 15 countries in this field. The USA ranks first with 1,258 publications (17.1%), followed closely by China with 1,257 publications (17.1%). This leading position of the USA and China may be attributed to their substantial investment in biomedical research, robust academic infrastructures, and extensive international collaboration networks. Notably, the USA outperforms in research impact, evidenced by a higher average citation per article (21.9) compared to China (10.9), reflecting the influence and quality of contributions from USA institutions. Japan ranks third with 781 publications (10.6%). In terms of collaboration types, China leads with 1,131 single-country publications (SCP), while the USA ranks first with 189 multiple-country publications (MCP). Additionally, Canada has the highest MCP ratio (32.4%), followed by Italy (20.6%). In terms of total citations (TC), the USA is the leader with 27,613 citations, followed by China (13,728 citations) and Japan (9,351 citations). The Netherlands has the highest average citations per article (25.40), followed by the USA (21.90) and Germany (21.80), indicating the substantial research impact of these countries.

**Table 1:** Top 15 Countries in publications on bone tumor imaging research.

Country	Articles	Articles %	SCP	MCP	MCP %	TC	Average Article Citations
USA	1258	17.1	1069	189	15	27613	21.90
CHINA	1257	17.1	1131	126	10	13728	10.90
JAPAN	781	10.6	747	34	4.4	9351	12.00
INDIA	506	6.9	471	35	6.9	4330	8.60
KOREA	332	4.5	311	21	6.3	4824	14.50
ITALY	311	4.2	247	64	20.6	5091	16.40
TURKEY	305	4.2	296	9	3	2722	8.90
GERMANY	275	3.7	223	52	18.9	5991	21.80
UNITED KINGDOM	267	3.6	203	64	24	4030	15.10
FRANCE	261	3.6	217	44	16.9	5221	20.00
CANADA	111	1.5	75	36	32.4	1606	14.50
IRAN	109	1.5	91	18	16.5	1454	13.30
BRAZIL	100	1.4	82	18	18	978	9.80
NETHERLANDS	100	1.4	84	16	16	2539	25.40
GREECE	82	1.1	70	12	14.6	898	11.00

Articles: Publications of Corresponding Authors only. SCP, Single-Country Publications; MCP, Multiple-Country Publications; TC, Total citations.

**Figure 3:**
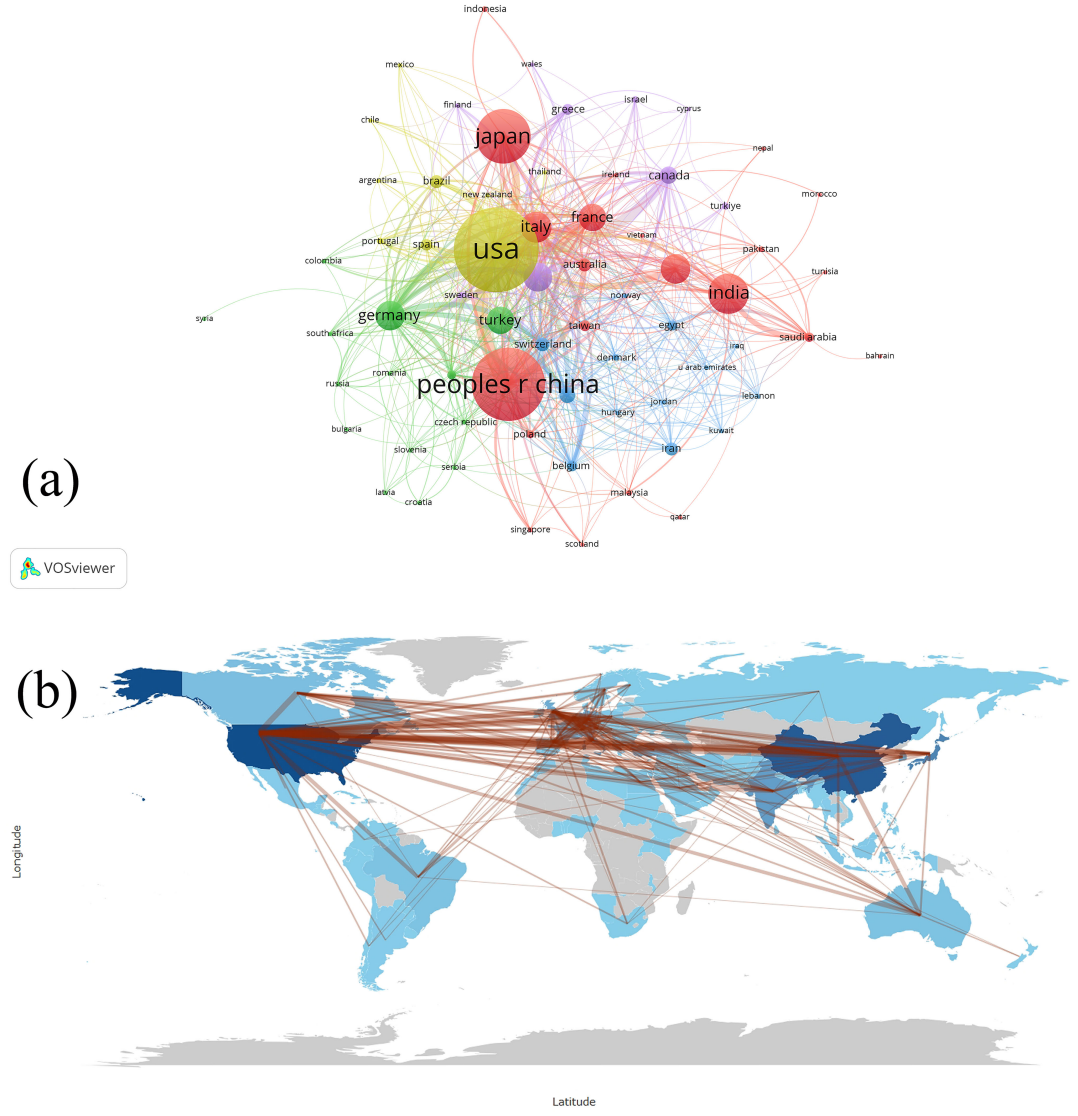
**(a)** Visualization map depicting the collaboration among different countries based on VOSviewer. Nodes represent countries, with size indicating publication count. Links represent co-authorships, with thickness showing collaboration strength. Colors indicate different research clusters. Total link strength in collaboration networks measures the frequency of co-authorship between countries, indicating the level of collaborative research. **(b)** Countries’ collaboration world map.

**Figure 3** depicts the international collaboration patterns in the field of bone tumor imaging research. **Figure 3a** focuses on the 68 countries that have published at least five articles. The USA, China, and Japan are positioned prominently, indicating their significant role in global research output. The total link strength between countries measures the frequency of co-authorships, emphasizing the interconnectedness of research efforts. **Figure 3b** presents a world map depicting the geographical distribution of these collaborations, with lines connecting countries based on their co-authorships. The map shows a high level of collaboration between countries in North America, Europe, and Asia, particularly between the USA, China, and Japan. This global network of collaboration highlights the international scope and cooperative nature of research in the field of bone tumors and medical imaging.

### 3.3 Analysis of institutions

**Figure 4:**
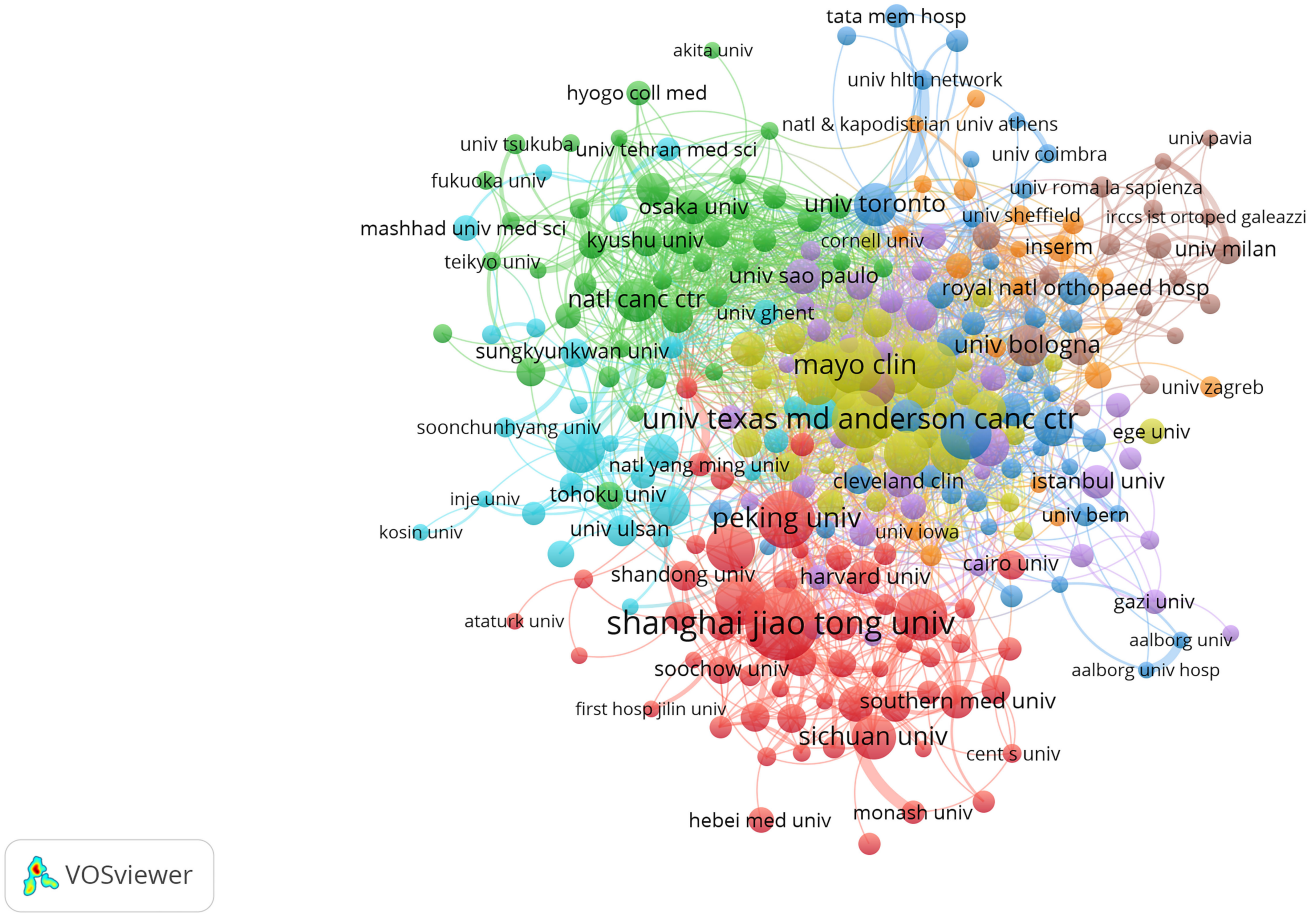
Visualization map depicting the collaboration among different institutions. Nodes represent institutions, with size indicating publication count. Links represent co-authorships, with thickness showing collaboration strength. Colors indicate different research clusters. Total link strength in collaboration networks measures the frequency of co-authorship between institutions, indicating the level of collaborative research.

**Table 2** presents the top 10 institutions contributing to bone tumor imaging research. The University of Texas System ranks first with 268 publications, followed by Harvard University (239 publications) and the University of California System (237 publications), indicating their leading roles in advancing the field. **Figure 4** provides a visualization of the collaboration networks among institutions involved in bone tumor imaging research, based on the analysis of 6,792 institutions worldwide. The figure highlights the 281 institutions that have published at least 10 articles in this field. The total link strength measures the frequency of co-authorships, providing insight into the level of international collaboration in bone tumor imaging research.

**Table 2:** Top 10 institutions in publications on bone tumor imaging research.

Institution	Articles
UNIVERSITY OF TEXAS SYSTEM	268
HARVARD UNIVERSITY	239
UNIVERSITY OF CALIFORNIA SYSTEM	237
SHANGHAI JIAO TONG UNIVERSITY	210
UNICANCER	198
UTMD ANDERSON CANCER CENTER	198
UNIVERSITY OF LONDON	184
UNIVERSITY OF TORONTO	181
SEOUL NATIONAL UNIVERSITY (SNU)	152
ASSISTANCE PUBLIQUE HOPITAUX PARIS (APHP)	149

### 3.4 Analysis of journals

**Table 3** presents the top 10 journals in bone tumor imaging research, detailing their impact and contribution to the field. *Skeletal Radiology* leads the list with 232 publications (NP) and a total of 2,761 citations (TC), indicating strong productivity and citation impact, supported by an h-index of 26 and a g-index of 36. *Cureus Journal of Medical Science* ranks second in publication volume with 136 papers, although its citation metrics are relatively modest, with an h-index of 6 and TC of 169. *Medicine* follows with 92 publications and moderate citation performance (TC: 396, h-index: 9). In terms of total citations, *Skeletal Radiology* still tops the list (TC: 2,761), followed by *European Radiology* (TC: 2,645), which also boasts the highest h-index (32) and g-index (47) among the top 10, reflecting its substantial academic influence. *European Journal of Radiology*, with 2,541 citations, ranks third in total citations and contributes significantly to the field with an impressive g-index of 46 and a Q1 JCR ranking.

**Table 3:** Top 10 journals in publications on bone tumor imaging research.

Source	h-index	g-index	m-index	TC	NP	IF	JCR 2023	PY_ start
*Skeletal Radiology*	26	36	0.929	2761	232	1.9	Q2	1998
*Cureus Journal of Medical Science*	6	7	0.6	169	136	1	Q3	2016
*Medicine*	9	15	0.818	396	92	1.4	Q2	2015
*European Journal of Radiology*	30	46	1.071	2541	91	3.2	Q1	1998
*International Journal of Surgery Case Reports*	10	15	0.714	340	90	0.6	Q4	2012
*European Radiology*	32	47	1.455	2645	85	4.7	Q1	2004
*Frontiers in Oncology*	12	15	0.923	367	73	3.5	Q2	2013
*Oncology Letters*	11	14	0.688	452	68	2.5	Q3	2010
*Clinical Orthopaedics and Related Research*	23	35	0.885	1334	58	4.4	Q1	2000
*Journal of Bone Oncology*	12	21	0.857	524	54	3.1	Q2	2012

The h-index of the journal, which measures both the productivity and citation impact of the publications (29). The g-index is capable of more accurately reflecting the contribution of highly cited papers in a journal (30). The m-index, by dividing the h-index by the number of years since the journal’s inception, eliminates the influence of time, allowing for a fairer comparison between journals of different founding years (31). TC, Total Citations; NP, Number of Publications; IF, Impact Factor, indicating the average number of citations to recent articles published in the journal; JCR_Quartile, The quartile ranking of the journal in the Journal Citation Reports, indicating the journal’s ranking relative to others in the same field; PY_start, Publication Year Start indicates the year the journal started publication.

### 3.5 Analysis of authors

**Figure 5:**
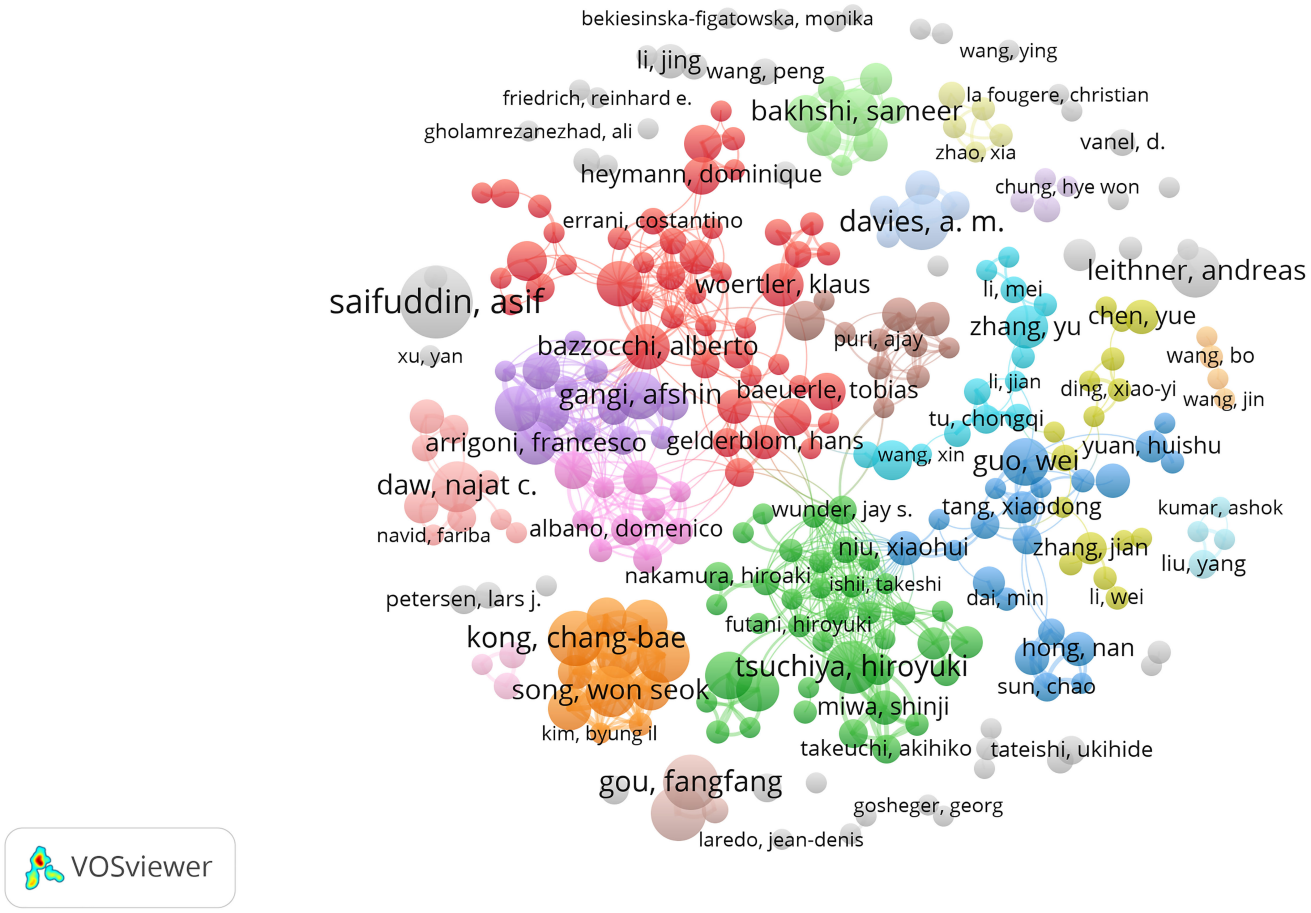
Visualization map depicting the collaboration among different authors. Nodes represent authors, with size indicating publication count. Links represent co-authorships, with thickness showing collaboration strength. Colors indicate different research clusters. Total link strength in collaboration networks measures the frequency of co-authorship between authors, indicating the level of collaborative research.

A total of 31,591 authors have contributed to bone tumor imaging research. Among these, Asif Saifuddin from the United Kingdom is the most prolific author, with 26 publications in the field, followed closely by A. M. Davies and Fangfang Gou, who have each published 19 articles. **Figure 5** illustrates the collaboration network among the 278 authors who published at least six articles on bone tumor imaging. The total link strength in the collaboration network measures the frequency of co-authorship between authors, highlighting the key figures in terms of research collaboration. Notably, Chang-Bae Kong (with a total link strength of 111), Wan Hyeong Cho (107), and Won Seok Song (99) emerge as the top collaborators, indicating their central role in fostering international and interdisciplinary partnerships in this area. The high link strength of these authors suggests significant contributions to collaborative research, underlining the importance of global cooperation in advancing the field of bone tumor imaging.

### 3.6 Analysis of keywords

**Figure 6:**
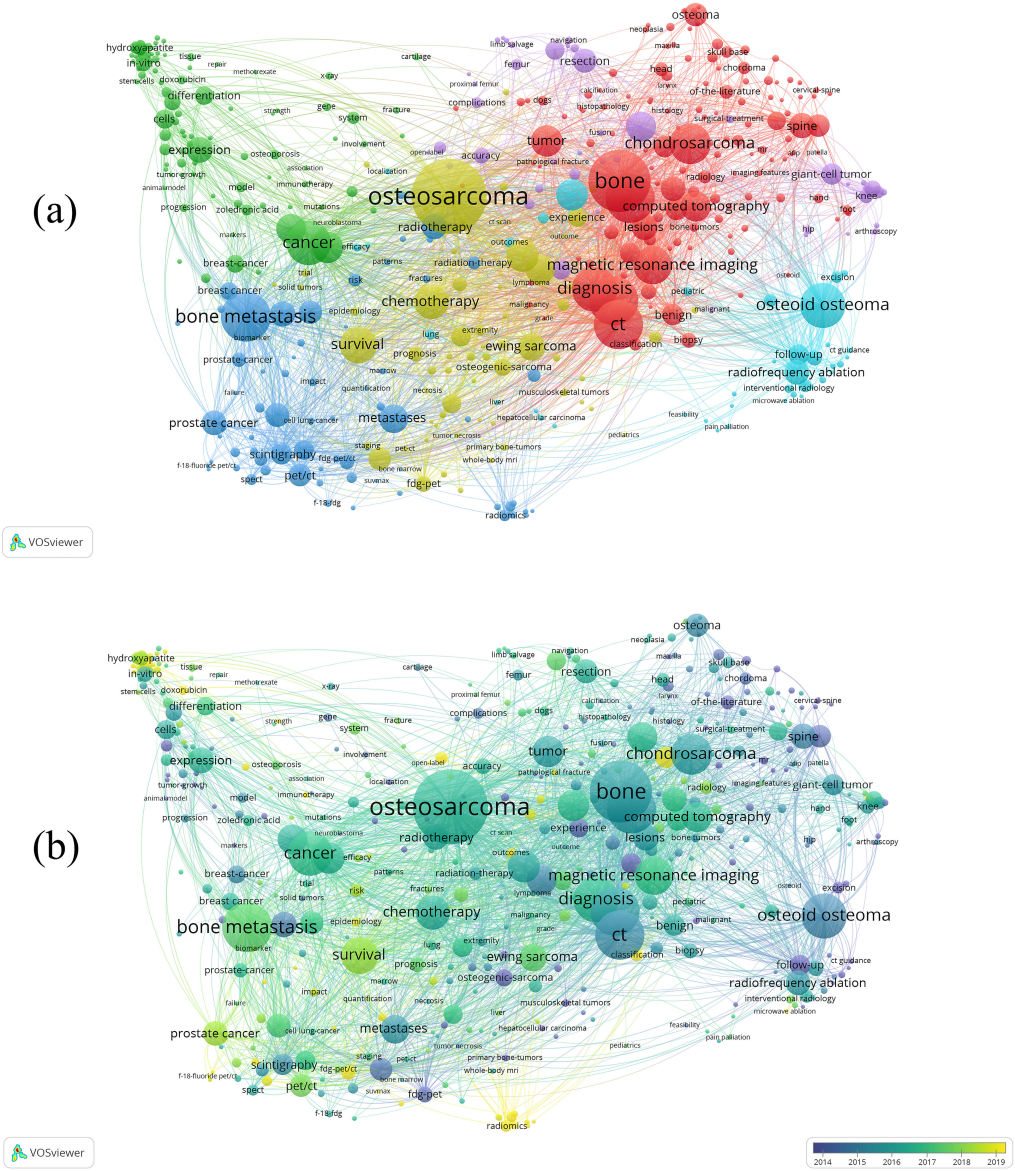
**(a)** Visual analysis of keyword co-occurrence network analysis. This network visualization displays the co-occurrence of keywords in selected literature. Each node represents a keyword, with size indicating its frequency of occurrence. Links between nodes represent co-occurrence in the same documents, with thicker lines showing stronger associations. Colors indicate different research clusters. **(b)** Time-overlapping co-occurrence analysis network of keywords. Colors reflect the average publication year of the articles, as indicated by the color gradient at the bottom right.

The bibliometric analysis of bone tumor imaging research involves more than 10,000 unique keywords, with **Figure 6** providing a detailed visualization of the co-occurrence network for the 454 keywords that appeared at least 20 times in the selected literature. **Table 4** lists the top 30 keywords, highlighting those with the most occurrences and total link strength, such as osteosarcoma, bone, bone metastasis, and CT. Among these, osteosarcoma (974 occurrences, 4,752 total link strength) stands out as the dominant keyword in this field, followed by bone (754 occurrences) and bone metastasis (561 occurrences), demonstrating their central role in bone tumor imaging research. In **Figure 6a**, the co-occurrence network is further distinguished by different color clusters, which represent various research themes and subfields within bone tumor imaging. Each color group reflects a distinct area of focus, with clusters such as those centered around osteosarcoma, bone metastasis, and diagnosis. These color-coded clusters help visualize the major themes in the field, such as imaging techniques (e.g., CT, MRI), treatment strategies (e.g., chemotherapy, radiotherapy), and tumor-specific research (e.g., osteoid osteoma, chondrosarcoma). The links between these keywords reveal the interconnectedness of various aspects of bone tumor imaging, reflecting a multidisciplinary approach to the research. **Figure 6b** shows the time-overlapping co-occurrence network, this analysis highlights recent shifts in research trends. Keywords like PET/CT and Radiomics have gained prominence in recent years, indicating their increasing relevance as cutting-edge technologies in bone tumor diagnosis and treatment.

**Table 4:** Top 30 keywords in publications on bone tumor imaging research.

Keyword	Occurrences	Total Link Strength
osteosarcoma	974	4752
bone	754	3759
bone metastasis	561	2532
ct	552	2917
mri	545	2816
tumors	544	2818
osteoid osteoma	491	2153
diagnosis	482	2540
cancer	481	2351
chondrosarcoma	430	1933
magnetic resonance imaging	387	1951
survival	369	2200
chemotherapy	336	1941
sarcoma	307	1637
management	296	1477
tumor	295	1484
therapy	294	1644
surgery	281	1401
computed tomography	278	1358
metastasis	276	1337
children	271	1516
metastases	263	1597
ewing sarcoma	244	1357
lesions	232	1322
expression	229	1049
radiotherapy	224	1168
carcinoma	223	1086
spine	219	1018
bone tumor	215	933
prostate cancer	213	1032

**Figure 7:**
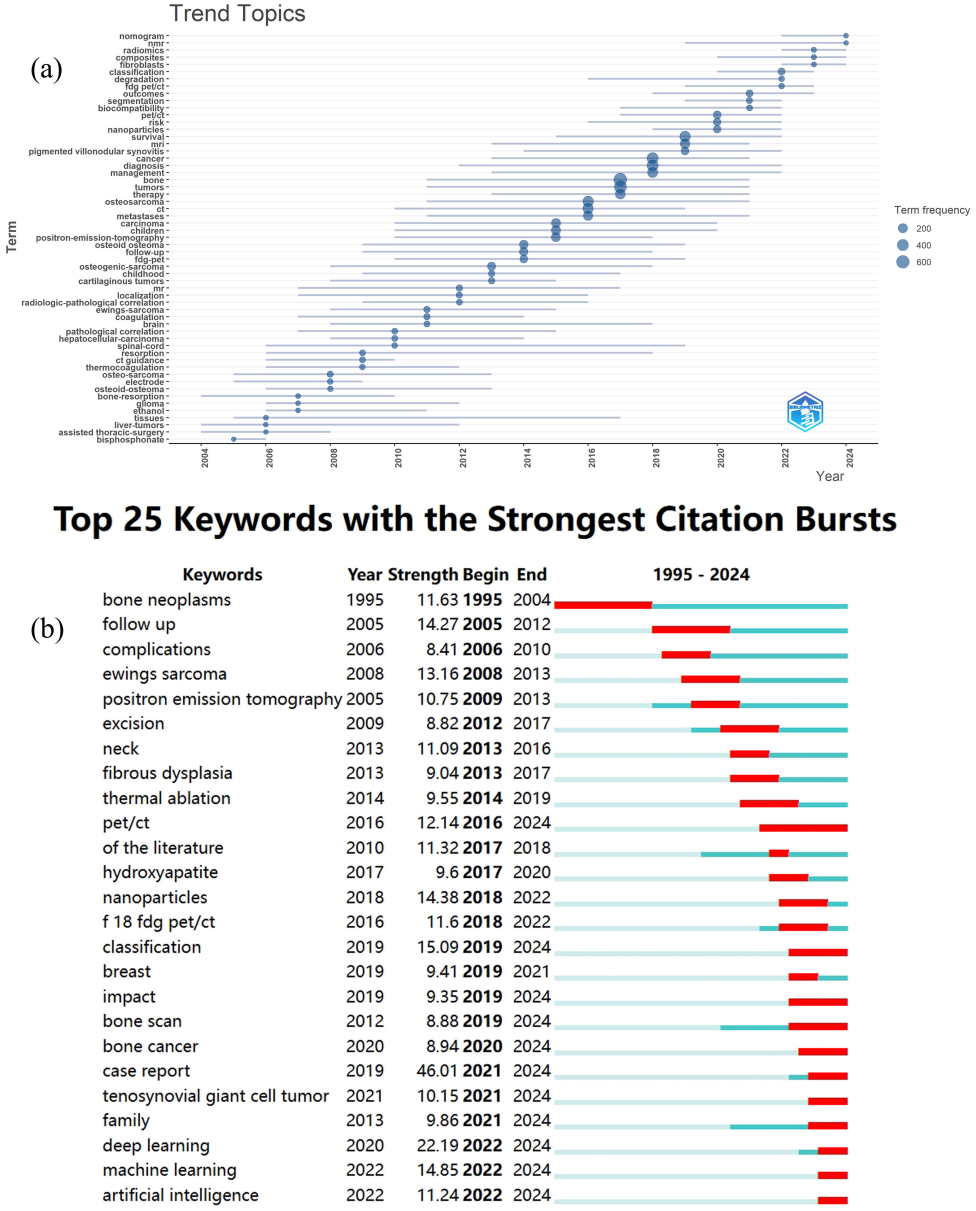
**(a)** The evolution of keyword frequency by Bibliometrix R. **(b)** The top 25 keywords with the strongest citation bursts by CiteSpace.

**Figure 7** provides an in-depth analysis of keyword trends in bone tumor imaging research, with insights into the evolution of keyword frequency and the keywords with the strongest citation bursts. **Figure 7a** presents the temporal evolution of keyword frequency, highlighting the gradual rise of key terms in the field. Notably, recent years have seen the emergence of keywords such as composites, radiomics, fibroblasts, nomogram, and NMR as significant areas of research. These keywords indicate a shift towards advanced methodologies in imaging and analysis, with radiomics and nomograms reflecting the increasing interest in extracting quantitative features from medical images and applying predictive modeling techniques to enhance diagnostic accuracy. The rise of fibroblasts and NMR suggests a growing focus on molecular and cellular mechanisms in bone tumor research, alongside traditional imaging approaches.

In **Figure 7b**, the top 25 keywords with the strongest citation bursts are displayed, shedding light on recent research breakthroughs. Among these, PET/CT, classification, impact, bone scan, bone cancer, case report, tenosynovial giant cell tumor, family, deep learning, machine learning, and artificial intelligence are highlighted as keywords with citation bursts extending into 2024. These keywords reflect the current research hotspots in the field. PET/CT continues to be a pivotal tool for detecting bone metastases and assessing treatment responses, while classification has become essential in the context of diagnosing and differentiating between various bone tumors. The growing focus on deep learning, machine learning, and artificial intelligence demonstrates the increasing role of computational techniques in automating image analysis and improving diagnostic precision. Bone cancer, case reports, and tenosynovial giant cell tumor represent ongoing areas of investigation into specific tumor types, while impact and bone scan highlight the importance of evaluating treatment outcomes and diagnostic methods. These recent developments underscore the shift towards integrating advanced imaging technologies with artificial intelligence and machine learning, positioning these topics as the cutting-edge trends in bone tumor imaging research.

## 4 Discussion

### 4.1 Overview of publication trends

The bibliometric analysis presented in this study offers a comprehensive view of the global research trends in bone tumors within the domain of medical imaging. The analysis period from 1995 to 2024 reveals a significant upward trend in publications, with a notable surge in research activity in the last decade. The peak in publications in 2024 highlights the current prominence of bone tumor research and medical imaging as pivotal areas of scientific inquiry.

The USA and China have emerged as the leading contributors to this research domain, with the highest volume of publications originating from institutions in these regions. Notably, the USA has maintained a dominant position, both in terms of publication quantity and quality, followed closely by China, which has shown rapid progress in recent years, likely driven by the country’s growing research infrastructure and healthcare advancements. Institutional analysis highlights the exceptional role of top research universities and specialized medical centers in driving innovation. The University of Texas System, Harvard University, and the University of California System are the top three institutions publishing in the field of bone tumor imaging, further emphasizing the leadership of the USA in this domain. *Skeletal Radiology*, as the journal with the highest number of publications on bone tumor imaging, plays a pivotal role in advancing research in this field. The author analysis indicates that Asif Saifuddin is the most prolific author in this area, with his recent study discussing the accuracy of chemical shift imaging in evaluating bone tumors (32), this work has contributed to the application of medical imaging technologies in the diagnosis of bone tumors.

### 4.2 Current research status

Medical imaging has become an indispensable tool in the diagnosis, staging, and monitoring of bone tumors. The evolving field of bone tumor research relies heavily on medical imaging techniques, and this shift toward imaging-based diagnostics underscores the centrality of these technologies in improving clinical outcomes and enhancing the precision of diagnosis.

Historically, the primary focus of bone tumor research in the realm of medical imaging has centered on the optimization and application of traditional imaging modalities such as CT and MRI (33). These imaging modalities have been critical in detecting bone tumors, determining their location, and assessing their extent. For instance, MRI is widely regarded as the preferred modality for imaging soft tissue tumors due to its superior contrast resolution (34), while CT is often utilized to assess bone involvement and detect structural changes (35). Keywords such as “MRI,” “CT,” and “radiology” have consistently appeared in the literature, indicating the continuing significance of these imaging techniques.

In addition to these well-established imaging modalities, the management of bone metastases has been a major research hotspot. Bone metastasis is one of the most common complications in patients with advanced cancers, particularly from primary tumors in the breast, prostate, lung, and kidney (36–38). Keywords such as “bone metastasis,” “metastatic bone disease,” and “skeletal metastases” reflect the substantial body of literature addressing the detection and management of metastatic bone tumors. Imaging plays a crucial role in identifying bone metastases early, monitoring their progression, and assessing their response to therapies. Techniques like whole-body MRI, bone scintigraphy, and PET/CT have been particularly important in the detection of bone metastases, enabling clinicians to evaluate both the number and location of metastatic lesions (14). In particular, PET/CT has become a significant tool for identifying bone metastases due to its high sensitivity and specificity in evaluating tumor spread and treatment response (39). At the same time, Radiomics is emerging as a novel approach to extracting high-dimensional quantitative features from medical images, which contributes to more precise diagnoses, individualized treatment planning, and improved prognostic assessment (40). These advancements underscore the growing role of advanced imaging and data-driven techniques in modern bone tumor research.

### 4.3 Emerging trends and future directions

Recent trends in bone tumor research, as reflected in our keyword analysis, highlight innovations in imaging modalities and targeted therapies. These trends reflect a move towards greater accuracy, personalization, and integration of advanced technologies. Below, we categorize these trends and discuss their potential implications for the future of bone tumor management.

#### 4.3.1 Multimodal imaging for enhanced diagnosis and monitoring

A major shift in recent research is the integration of multimodal imaging techniques to improve diagnostic and prognostic capabilities. The combination of PET/CT, PET/MRI, and other imaging modalities allows for both anatomical and functional assessments of bone tumors, offering a more comprehensive understanding of tumor behavior. For example, Nappi’s study showed that PET/CT has a high sensitivity and specificity for the assessing marrow involvement in pediatric solid tumors (41). Xia’s research indicated that [F-18]FDG PET/MRI demonstrates superior sensitivity and similar specificity to [F-18]FDG PET/CT in detecting bone metastases in breast cancer patients (42). The integration of these modalities enables clinicians to better stage bone tumors, detect early metastases, and monitor therapeutic responses. As imaging technologies continue to evolve, we expect further advancements in multimodal imaging approaches. The development of hybrid imaging systems that combine molecular imaging with high-resolution MRI or CT will enable deeper insights into tumor microenvironments, thus enhancing early detection, treatment monitoring, and even prediction of treatment responses.

#### 4.3.2 AI and machine learning in imaging

AI and machine learning have made significant inroads in medical imaging, particularly in the analysis of large datasets. In bone tumor research, the use of AI algorithms to analyze imaging data has shown great promise in automating image interpretation, identifying tumor characteristics, and predicting patient outcomes. Keywords like “deep learning,” “radiomics,” and “machine learning” have emerged as key trends in recent publications. For example, Wang’s research showed that radiomics-clinical nomogram has high accuracy in identifying bone metastasis and benign bone diseases in cancer patients (43). Li’s team developed a lightweight convolutional neural network model for automatic recognition of bone tumor pathological images, which achieved an accuracy of 99.06% in bone tumor classification (44). Deng’s team developed an efficient and accurate machine learning model for the diagnosis of primary bone tumors (45). These results indicate that the integration of AI with imaging not only enhances diagnostic precision and reduces observer variability but also shortens the time needed for image interpretation. In clinical practice, such improvements facilitate earlier detection of malignant lesions, thereby enabling timely clinical interventions that may lead to improved patient survival and quality of life (46). The continued integration of AI and machine learning with imaging will lead to the development of personalized diagnostic models, where algorithms can analyze individual patient data to tailor treatment strategies. AI can potentially identify imaging biomarkers that predict patient responses to specific therapies, further enhancing precision oncology for bone tumors.

#### 4.3.3 Molecular and functional imaging for tumor characterization

Molecular imaging and functional imaging have emerged as critical areas of interest in bone tumor research. Fluorescence imaging has been successfully used to detect bone tumor remnants and optical diagnostic imaging of bone metastases (47, 48). Dynamic contrast-enhanced-MRI (DCE-MRI) has been found useful for the characterization of bone tumors (49). Additionally, whole-body diffusion-weighted MRI (DW-MRI) can produce a better visual distinction between healthy and diseased bone marrow, leading to better diagnosis (50). These functional imaging techniques provide more detailed information on tumor biology and are valuable for assessing response to treatment, particularly in metastatic bone disease. As imaging techniques evolve, they will enable clinicians to visualize and track the molecular targets of specific therapies.

#### 4.3.4 Bone metastasis and early detection of metastatic disease

Bone metastasis continues to be a key area of research, particularly with regard to early detection and monitoring disease progression. In recent years, a large number of studies on bone metastasis have emerged. For example, Young studied the mechanism of bone (re)modeling in breast cancer to bone metastasis (51). Jiang’s latest article discussed the early diagnostic value of emission computed tomography whole-body bone imaging for bone metastasis of lung cancer (52). The early identification of metastatic bone lesions can significantly influence treatment outcomes, as more aggressive therapies can be initiated earlier, improving survival rates. Advancements in functional imaging techniques such as PET and MRI, alongside emerging radiomic models, will enable earlier detection of bone metastasis, even in asymptomatic patients. The combination of these imaging tools with AI algorithms will also improve the sensitivity and specificity of bone metastasis detection, potentially reducing the need for invasive biopsies and offering a non-invasive alternative for regular monitoring.

### 4.4 Limitations

This study provides a comprehensive analysis of global research trends in bone tumor imaging; however, it still has certain limitations. For instance, the inclusion criteria were restricted to English-language articles, potentially excluding valuable research published in other languages. Additionally, the study focused solely on the WoSCC database, which may not encompass all relevant publications in the field. Excluding other databases could result in the underrepresentation of certain research areas or the omission of significant studies. Furthermore, this research only includes studies published up to 2024, and recent high-quality studies may not have received due attention due to citation delays; these should be updated in future research. Nevertheless, this study offers a comprehensive overview of research trends and hotspots in bone tumor imaging, providing clear insights for scholars in this field.

## 5 Conclusion

This bibliometric analysis provides a comprehensive overview of the global research landscape on bone tumors and medical imaging. Over the past few decades, the field has seen remarkable growth, driven by advancements in imaging technology and a growing focus on the molecular and functional aspects of bone tumors. The integration of multimodal imaging, particularly PET/CT and MRI, is increasingly recognized as a pivotal approach to detect and monitor both primary and metastatic bone tumors. Furthermore, artificial intelligence and machine learning are emerging as powerful tools to improve diagnostic accuracy, automate image analysis, and facilitate personalized treatment approaches.

## Data Availability

The raw data supporting the conclusions of this article will be made available by the authors, without undue reservation.
